# Diagnostic challenge and surgical management of Boerhaave’s syndrome: a case series 

**DOI:** 10.1186/s13256-021-03080-1

**Published:** 2021-11-08

**Authors:** Jiayue Wang, Degang Wang, Jianjiao Chen

**Affiliations:** 1 Department of Obstetrics and Gynecology, Affiliated Jinhua Hospital, Zhejiang University School of Medicine, Jinhua, Zhejiang Province China; 2 Department of Thoracic Surgery, Chaoyang Municipal Central Hospital, Chaoyang, Liaoning Province China; 3 Department of Gastrointestinal Surgery, Sun Yat-sen Memorial Hospital, Sun Yat-sen University, Guangzhou, Guangdong China

**Keywords:** Boerhaave’s syndrome, Esophageal perforation, CT, Surgical management

## Abstract

**Background:**

Boerhaave’s syndrome is the spontaneous rupture of the esophagus, which requires early diagnosis and treatment. Symptoms may vary, and diagnosis can be challenging.

**Case presentation:**

Case 1: A 54-year-old Chinese man presented to us with sudden-onset epigastric pain radiating to the back following hematemesis. Upper gastrointestinal endoscopy revealed a full-thickness rupture of the esophageal wall. Subsequent computed tomography showed frank pneumomediastinum and heterogeneous pleural effusion. Immediately, esophageal perforation repair operation and jejunostomy were performed. The postoperative period was uneventful, and he was discharged.

Case 2: A 62-year-old Chinese man was admitted to the emergency department with thoracic dull pain and chest distress. Chest computed tomography scan showed pneumomediastinum and large left-sided pleural effusion. Esophagus fistula was confirmed by contrast esophagography. Then, we performed thoracotomy to repair the esophageal tear as well as to debride and irrigate the left pleural space. His postoperative period was uneventful, with no leakage or stricture.

Case 3: The patient was a 69-year-old Chinese male presenting with severe retrosternal and upper abdominal pain following an episode of forceful vomiting. Thoracic computed tomography scan revealed a rupture in the left distal part of the esophagus, a pneumomediastinum, and left-sided pleural effusions. Conservative treatment failed to improve disease conditions. Open thoracic surgery was performed with debridement and drainage of the mediastinum and the pleural cavity, after which he made a slow but full recovery.

**Conclusions:**

We highlight that early diagnosis and appropriate surgical treatment are essential for optimum outcome in patients with esophageal rupture. We emphasize the importance of critical care support, particularly in the early stages of management.

## Introduction

Boerhaave’s syndrome is a life-threatening condition characterized by a disruption of the distal esophagus due to barotrauma that results in contamination of the mediastinum and pleural cavity with gastric contents [1]. Symptoms may vary, and diagnosis can be challenging, as the classic triad of Mackler (vomiting, lower thoracic pain, and subcutaneous emphysema) is present in a few cases [2]. A significant delay between perforation and treatment often leads to mediastinitis followed by septic shock and multiorgan failure [3]. For emergency physicians, what counts is chest radiology examination, in which almost all abnormalities are visible. Although there is no consensus on optimum treatment strategy, surgery gets first priority among all therapeutic approaches. Here we describe three typical cases of Boerhaave’s syndrome with representative clinical symptoms and imaging manifestations. We developed multimodal treatment strategies and achieved desired effect.

## Case 1

**Fig. 1:**
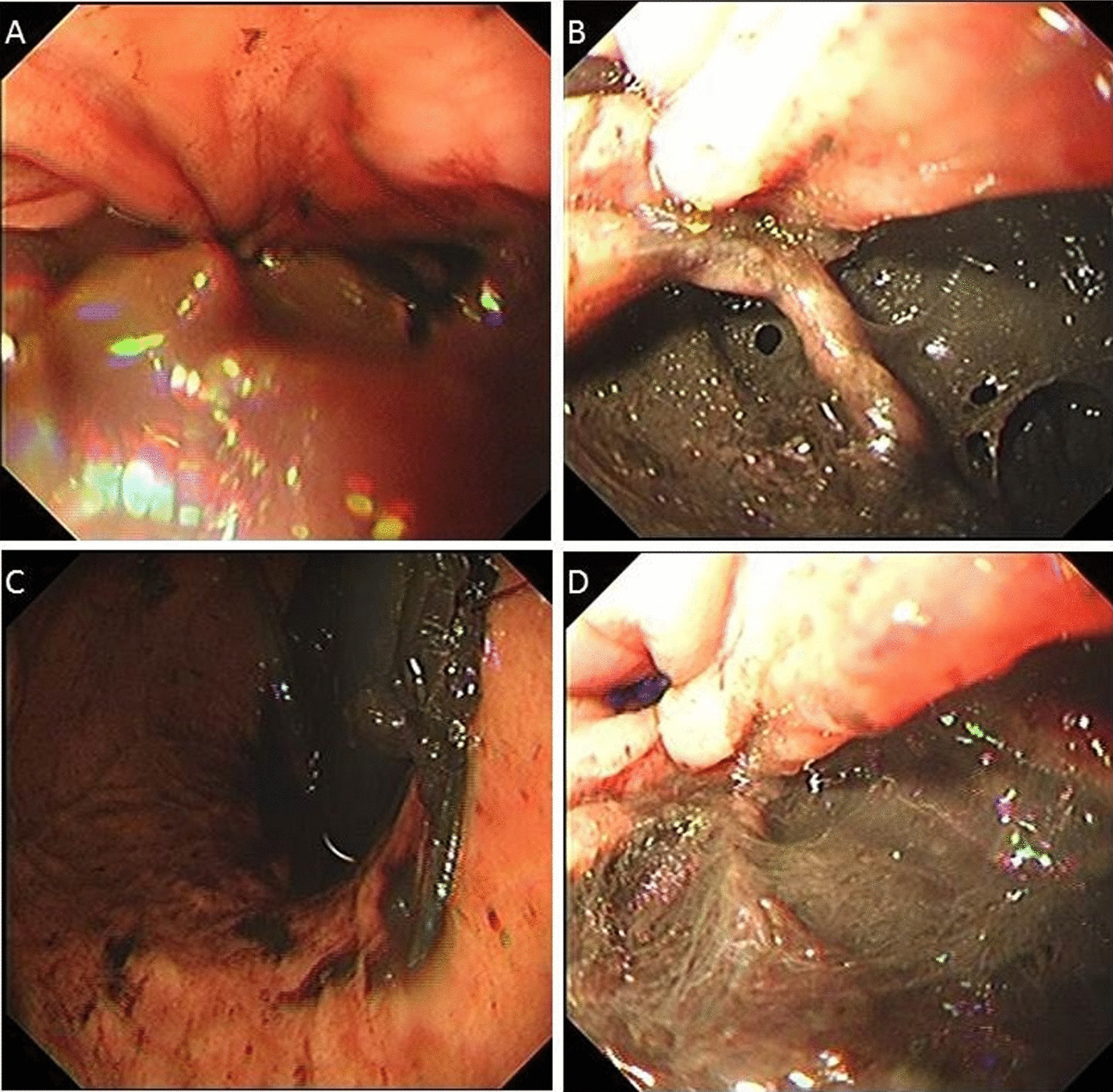
Upper gastrointestinal endoscopy revealing a full-thickness rupture of the esophageal wall

**Fig. 2:**
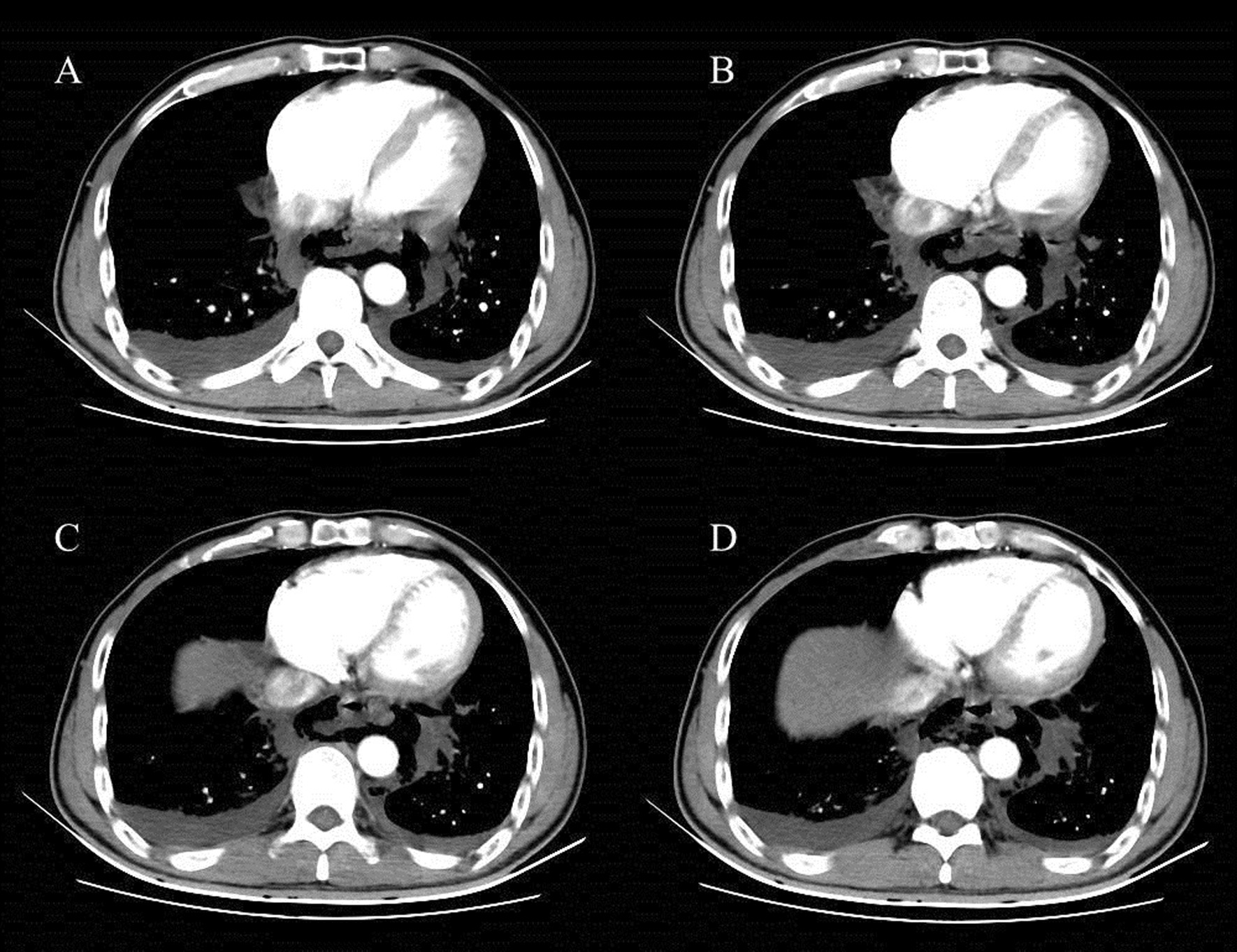
CT showing esophageal rupture with air leakage into the mediastinum and heterogeneous pleural effusion

A 54-year-old Chinese man with a previous medical history of gastric ulcer developed sudden-onset epigastric pain radiating to the back following hematemesis and presented to the emergency department. His blood pressure was 121/69 mmHg, pulse rate 110 beats/minute, and percutaneous oxygen saturation 93%. His axillary temperature was 37.2 °C. His laboratory results of routine blood tests were normal at the time of presentation. Initially, emergency doctor suspected a diagnosis of peptic ulcer with hematemesis, and esophagogastroscopy was performed. However, upper gastrointestinal endoscopy revealed a full-thickness rupture of the esophageal wall. Apart from this, the gastroesophageal mucosa was normal; in particular, there were no signs of ischemia (Fig. 1). Immediately, the patient underwent chest computed tomography (CT) scan, which showed frank pneumomediastinum and heterogeneous pleural effusion (Fig. 2). We considered the occurrence of spontaneous esophageal rupture known as Boerhaave’s syndrome. Therefore, the patient underwent esophageal perforation repair operation and jejunostomy. Postoperatively, the patient was transferred to the intensive care unit (ICU) for further conservative treatment. He recovered after a 25-day hospital stay, with feeding through jejunum nutrient catheter. Four months later, we removed his jejunum nutrient catheter.

## Case 2

**Fig. 3:**
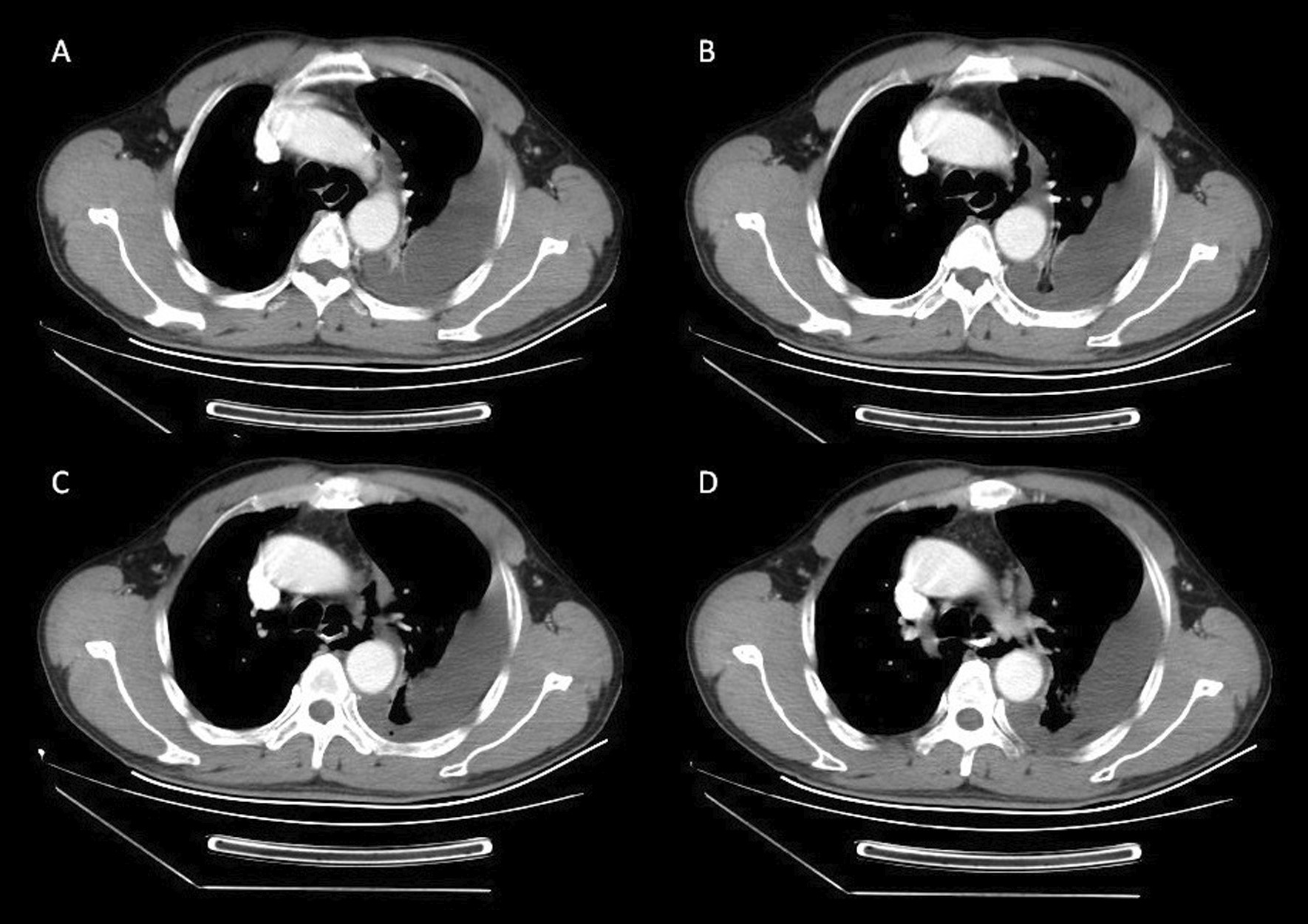
CT showing a tear in the left anterolateral wall of the distal esophagus, pneumomediastinum, and left pleural effusions

**Fig. 4:**
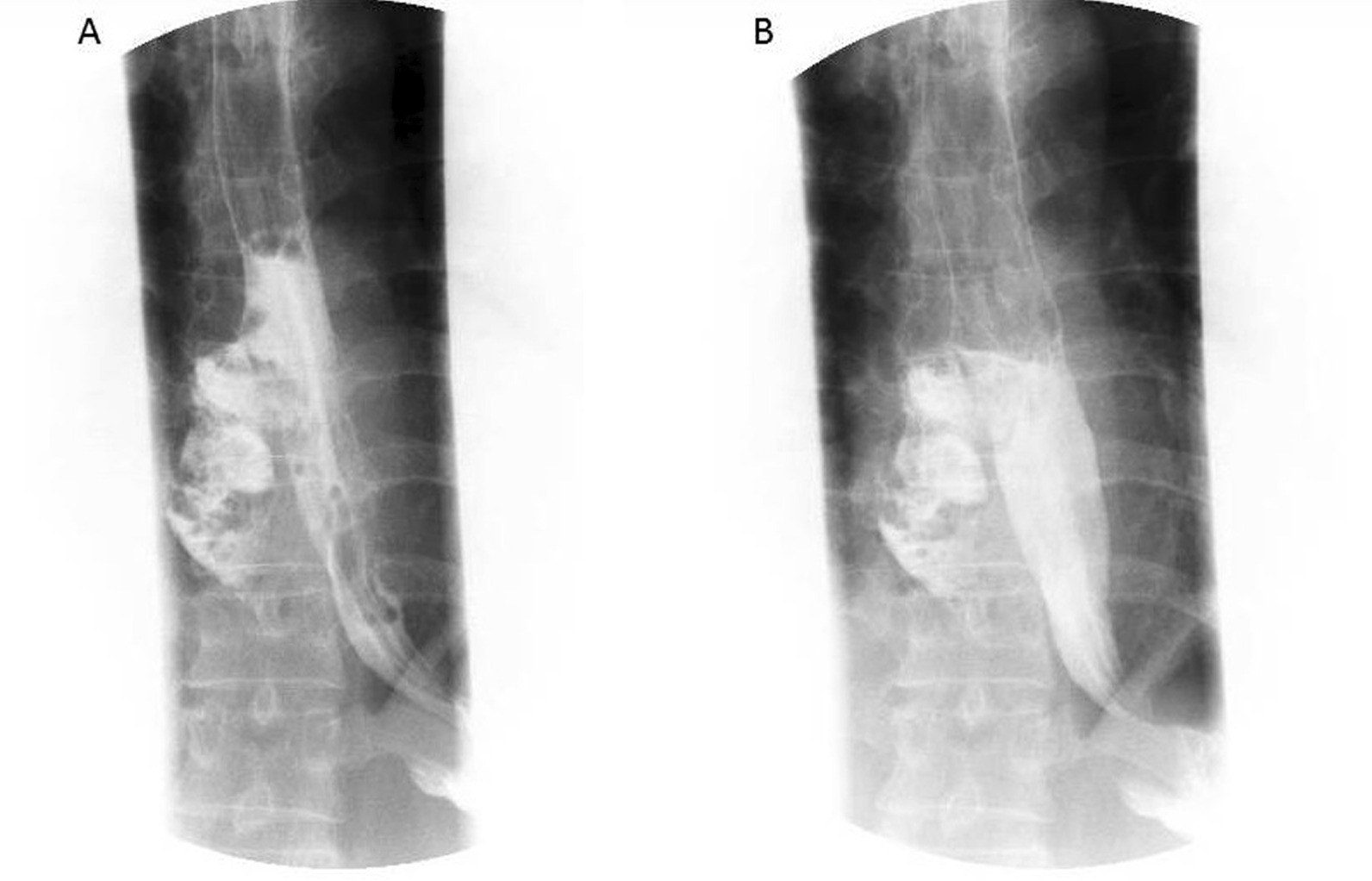
Esophagus fistula was further confirmed by contrast esophagography

A 62-year-old Chinese man was admitted to the emergency department with thoracic dull pain and chest distress that started after he had been vomiting several hours before presentation. At admission, his blood pressure was 116/78 mmHg, pulse rate was 100 beats/minute, percutaneous oxygen saturation was 96%, and axillary temperature was 36.5 °C. On physical examination, he presented rough bronchovesicular breathing sound, and crepitant rales in lungs prompting subcutaneous emphysema. His abdomen was smooth without tenderness or rebound pain. Laboratory results of routine blood tests and biochemical examination were normal at the time of presentation. A chest CT scan showed pneumomediastinum and large left-sided pleural effusion, and possible esophagus tear was noted (Fig. 3). Esophagus fistula was further confirmed by contrast esophagography (Fig. 4). Therefore, spontaneous esophageal perforation was suspected. As the patient’s general condition was progressively deteriorating, we immediately arranged emergency surgery for him. In consideration of narrow tearing site and scope, esophageal repair under endoscopy was not suitable. Thus, we performed thoracotomy to repair the esophageal tear as well as to debride and irrigate the left pleural space. Postoperatively, the patient was transferred to the intensive care unit for further treatment. A contrast esophagography finding obtained on postoperative day 12 demonstrated no sign of esophageal leak, and then he was started on an oral liquid diet. After an ICU stay of 20 days, the patient was discharged.

## Case 3

**Fig. 5:**
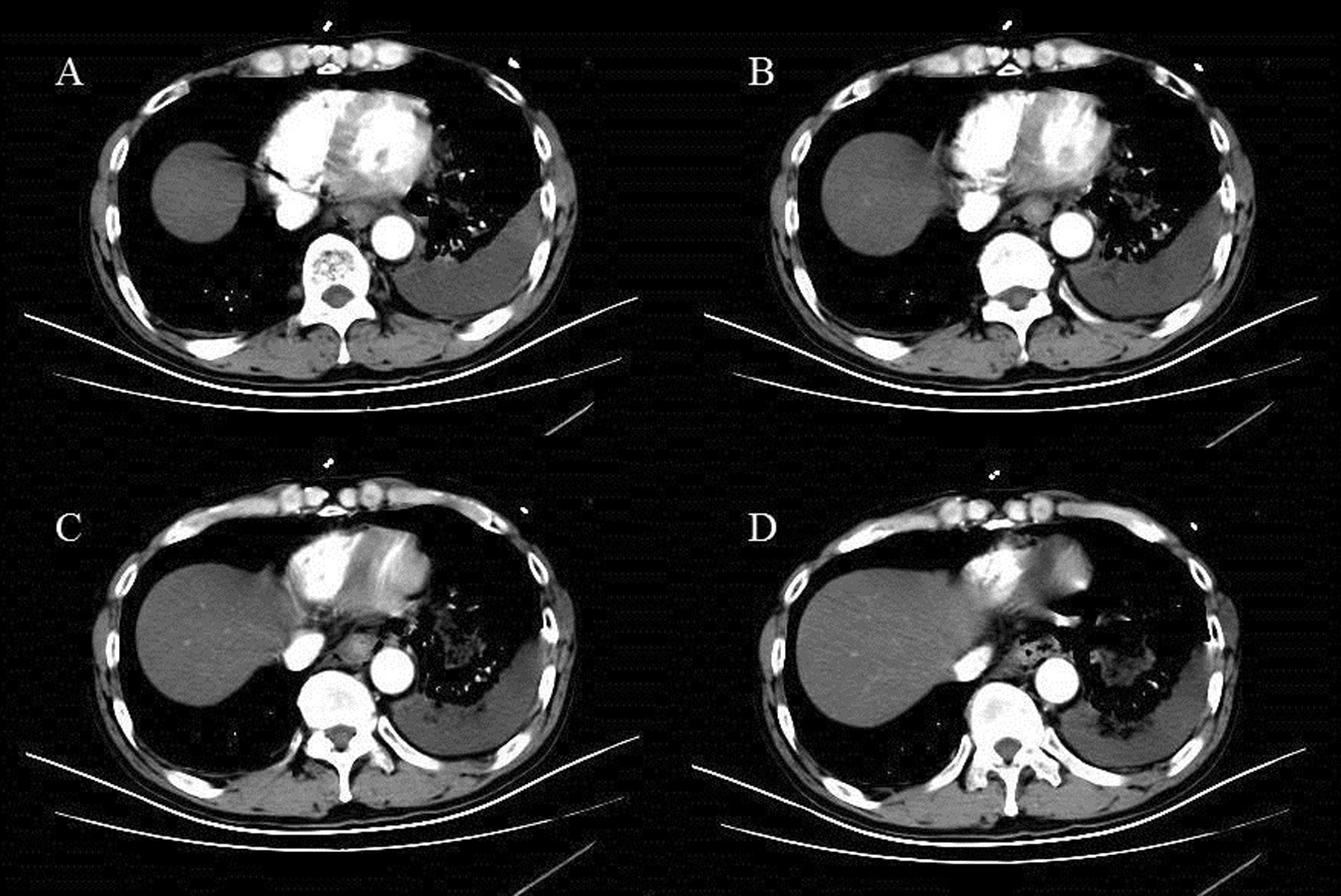
CT showing pneumomediastinum with air tracking laterally towards the left pleural space, and a large left pleural effusion

A 69-year-old Chinese man with a history of hypertension was referred to our emergency department with severe retrosternal and upper abdominal pain followed by an episode of forceful vomiting after he drank a bottle of beer at home 3 hours ago. At admission, he was diaphoretic and in respiratory distress. Blood pressure was 185/98 mmHg, pulse rate was 105 beats/minute, oxygen saturation was 94%, and core temperature was 36.3 °C. Physical examination revealed extensive cervical and thoracic subcutaneous emphysema, but was otherwise unremarkable. Laboratory results were normal by the time of presentation. A thoracic CT scan revealed a rupture in the left distal part of the esophagus, a pneumomediastinum, and left-sided pleural effusions (Fig. 5). Conservative treatment, with cessation of oral intake, nasogastric suction, administration of intravenous fluids and parenteral nutrition, intravenous broad-spectrum antibiotics, proton pump inhibitors, and drainage of the pleural effusion by left-sided thoracostomy, failed to improve disease conditions. Open thoracic surgery was performed with debridement and drainage of the mediastinum and the pleural cavity, after which he made a slow but full recovery.

## Discussion and conclusions

Boerhaave’s syndrome is defined as spontaneous esophageal rupture due to sudden increase in esophageal pressure accompanied by inability of the cricopharyngeal sphincter to relax [4]. It is a rare but life-threatening condition, for absence of mucous muscularity in esophagus allows food remains and gastric juices to enter the mediastinum, resulting in extensive infection of the mediastinum and pleural cavities without proper treatment.

Clinical manifestations at the disease’s earliest stages are nonspecific, depending on the site of esophageal perforation and time elapsed between its development and examination. Presence of Mackler triad (vomiting, lower thoracic pain, and subcutaneous emphysema) always prompts high possibility of irretrievability. It is essential to shorten the length of time between symptom manifestation and proper surgical treatment [5]. Imaging examination is of great significance for early diagnosis of Boerhaave’s syndrome. Chest radiology examination findings such as pleural effusion or pneumomediastinum are of great significance for diagnosis. Thoracic CT is helpful in differential diagnosis with other traumatic esophageal injury. Further contrast esophagography may provide exact evidence of contrast leakage from the esophageal lumen to make a definite diagnosis. Although endoscopy may provide direct vision evidence, we do not recommend endoscopy as a routine checking method because its invasiveness may deteriorate the lacerated condition. Early diagnosis and management (“golden early 24-h rule”) are crucial for benign outcome in patients with rupture of the esophagus.

The therapeutic choices for Boerhaave’s syndrome generally include conservative, surgical, and endoscopic treatments. The appropriate management of esophageal perforation is a controversial issue. Although there is as of yet no definitive treatment approach, according to current retrospective analysis and clinical experiences, application of operative intervention to repair breakage primarily is considered as priority in current management of Boerhaave’s syndrome, especially in a condition as we reported, with the patient presenting signs of contamination of the pleural cavity and mediastinum [6]. Traditional thoracotomy is still practical in emergency for its intuition. This surgical treatment is widely accepted as preferred treatment if tissues are viable.

It is worth noting that introduction of video-assisted thoracoscopic surgery (VATS) offers us more surgical choices for Boerhaave’s syndrome. Some clinicians have progressed zealously in application of VATS for Boerhaave’s syndrome and achieved beneficial results. Some clinicians have made positive attempts and achieved good results. A retrospective study comparing open thoracotomy or VATS for Boerhaave’s syndrome reported that VATS may be used as first choice for Boerhaave’s syndrome, since it is a safe procedure with a complication rate and results same as those for open thoracotomy [7]. For some lower esophageal perforation, laparoscopic surgery displays unique advantage over open surgery, which shows intraoperative precise positioning and enhanced recovery after surgery. Minimally invasive surgical management of Boerhaave’s syndrome is feasible and safe, with outcomes that compare favorably to the published literature [8].

Surgical treatment is well established, but the development of interventional endoscopy has proposed new therapies. Indeed, there are several case reports and publications in the literature regarding the endoscopic treatment of Boerhaave’s syndrome. Endoscopy is a proven effective method in the diagnosis of Boerhaave’s syndrome, particularly in cases in which the diagnosis is suspected but findings on CT are inconclusive, because endoscopy enables specific lesion characterization. Regarding endoscopic treatment, different therapeutic mechanisms can be proposed: closing with over-the-scope clips [9, 10], endoscopic ligation with snare loops [11], derivation indwelling esophageal stent [12, 13], and placement of open-pore polyurethane foam or luminal sponge [14, 15]. From the perspective of trauma degree, endoscopic management is reserved for treatment of inoperable patients. In spite of the minimal invasion, endoscopic treatment fails to debride and irrigate the polluted pleural space, which may present a potential risk in postoperative rehabilitation. By combining VATS and endoscopy, we can carry out the share of function of both treatments and achieve advantage complementarily. To the best of our knowledge, there is no report in the literature that describes application of combined VATS and endoscopy in a patient with Boerhaave’s syndrome. We look forward to application of this surgical management for Boerhaave’s syndrome.

Postoperative management is particularly crucial for Boerhaave’s syndrome patients. Anti-infection treatment and nutritional support are crucial in postoperative management [16]. Patients should be kept fasting per oral and should be placed on nasogastric tube to clear gastric secretory contents and limit further contamination. Intercostal chest tube would be a good choice for chest drainage, in surgical management as well as conservative treatment. Broad-spectrum antibiotics should be given as soon as possible and last for some time. By strengthening nutritional support, both primary and secondary esophageal leaks are being treated with reduced hospital stay and early resumption of oral diet. Repeated and regular contrast studies should be utilized to ascertain the progress of the treatment. For optimum outcome of management of esophageal perforations in adults, a multidisciplinary approach is needed.

## Data Availability

All data generated or analyzed during this study are included in this published article.
